# Supplementary figures and images for: Screening of differentially expressed immune-related genes from spleen of broilers fed with probiotic Bacillus cereus PAS38 based on suppression subtractive hybridization
Source: PLoS One. 2019 Dec 23;14(12):e0226829. doi: 10.1371/journal.pone.0226829 (PMC6927618; doi:10.1371/journal.pone.0226829)

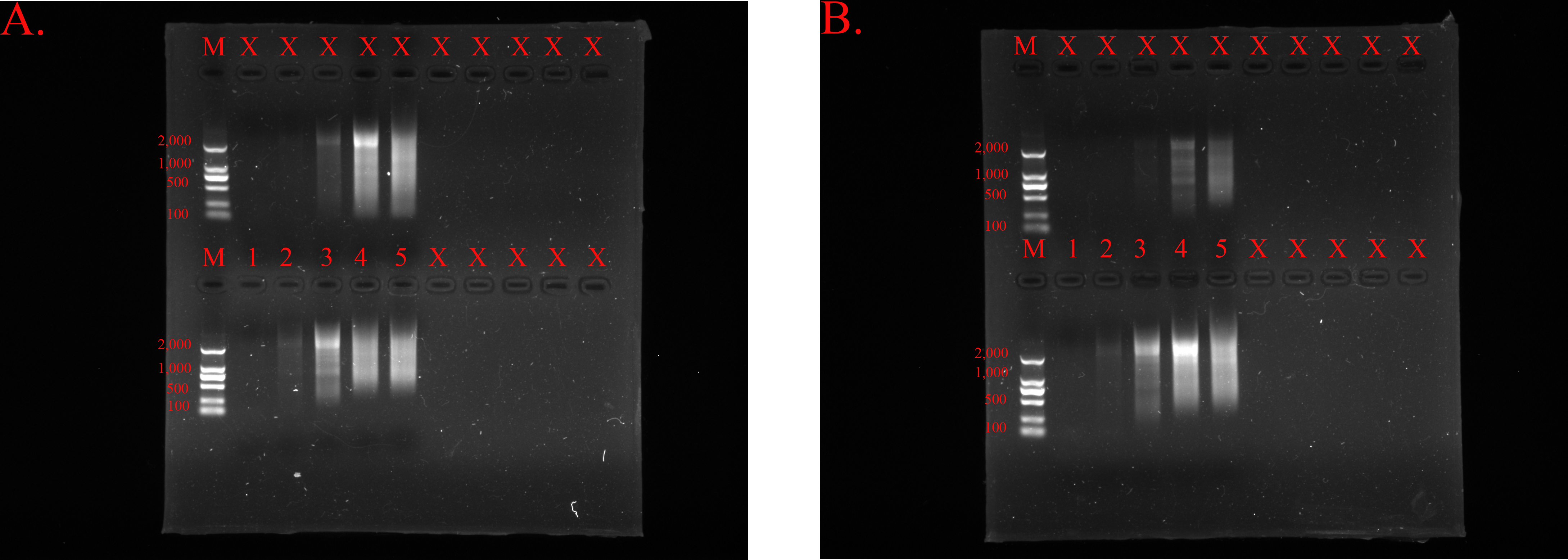

Supplement: S1 Fig — Electrophoresis with agarose of 1.2% concentration. The images were generated by the Gel imaging system Gel Doc™ XR+. (A) Treated group. (B) Control group. M represents DNA Marker 2000. The numbers 1, 2, 3, 4 and 5 represent 18, 21, 24, 27 and 30 PCR cycles respectively. Fig 1A was generated by S1A Fig, and Fig 1B was generated by S1B Fig. (TIF) [file pone.0226829.s001.tif]

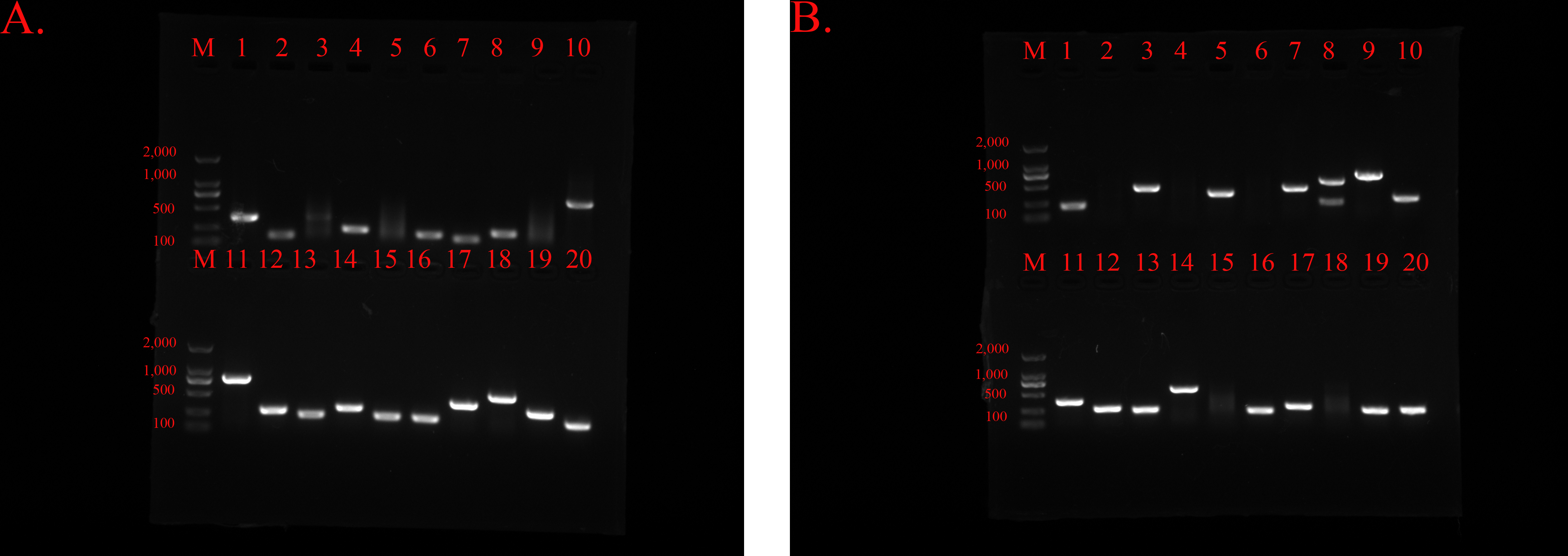

Supplement: S2 Fig — Electrophoresis with agarose of 1.2% concentration. The images were generated by the Gel imaging system Gel Doc™ XR+. (A) Treated group. (B) Control group. M represents DNA Marker 2000. The numbers 1, 2, 3, et al. represent different bacterial clones. Fig 2A was generated by S2A Fig, and Fig 2B was generated by S2B Fig. (TIF) [file pone.0226829.s002.tif]

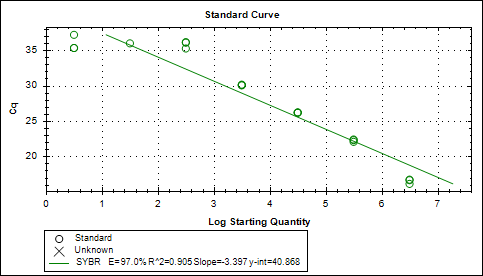

Supplement: S3 Fig — The abscissa represents the concentration of plasmid standard (Log10N copies/μL). The longitudinal coordinates denote the cycle threshold. The same below. (TIF) [file pone.0226829.s003.tif]

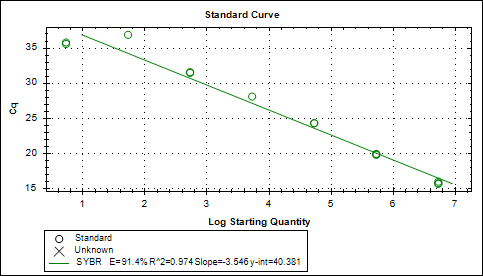

Supplement: S4 Fig — (TIF) [file pone.0226829.s004.tif]

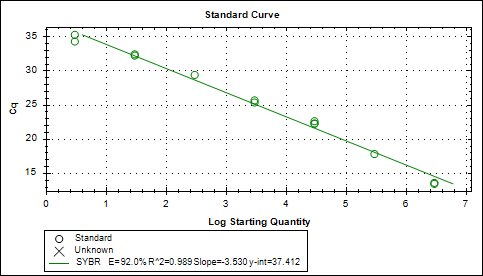

Supplement: S5 Fig — (TIF) [file pone.0226829.s005.tif]

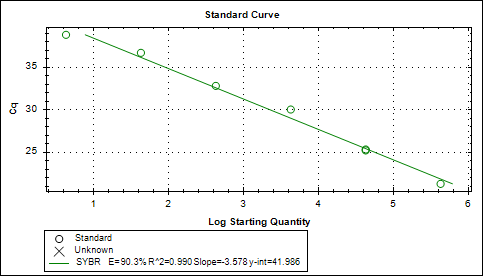

Supplement: S6 Fig — (TIF) [file pone.0226829.s006.tif]

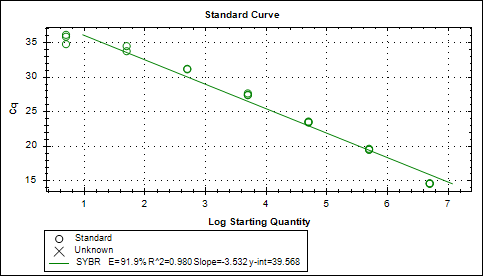

Supplement: S7 Fig — (TIF) [file pone.0226829.s007.tif]

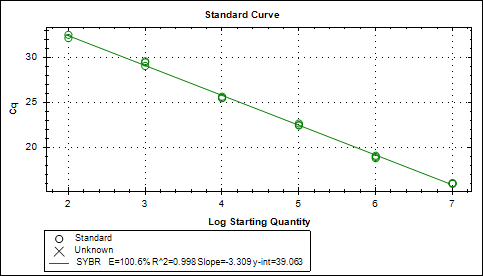

Supplement: S8 Fig — (TIF) [file pone.0226829.s008.tif]

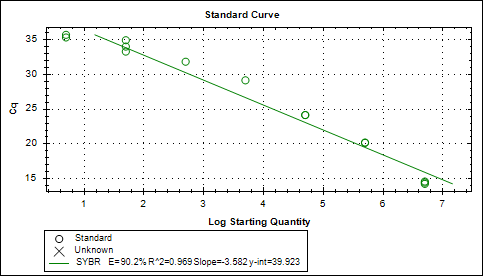

Supplement: S9 Fig — (TIF) [file pone.0226829.s009.tif]

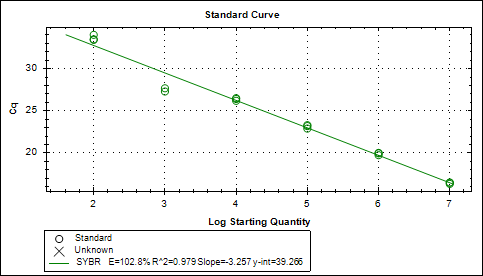

Supplement: S10 Fig — (TIF) [file pone.0226829.s010.tif]

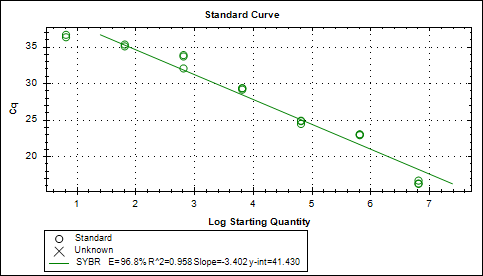

Supplement: S11 Fig — (TIF) [file pone.0226829.s011.tif]

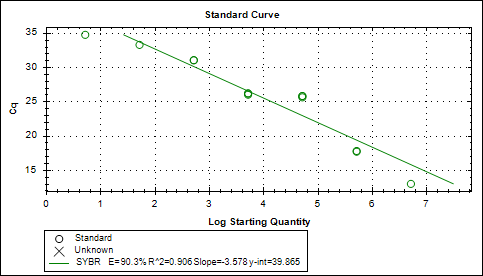

Supplement: S12 Fig — (TIF) [file pone.0226829.s012.tif]

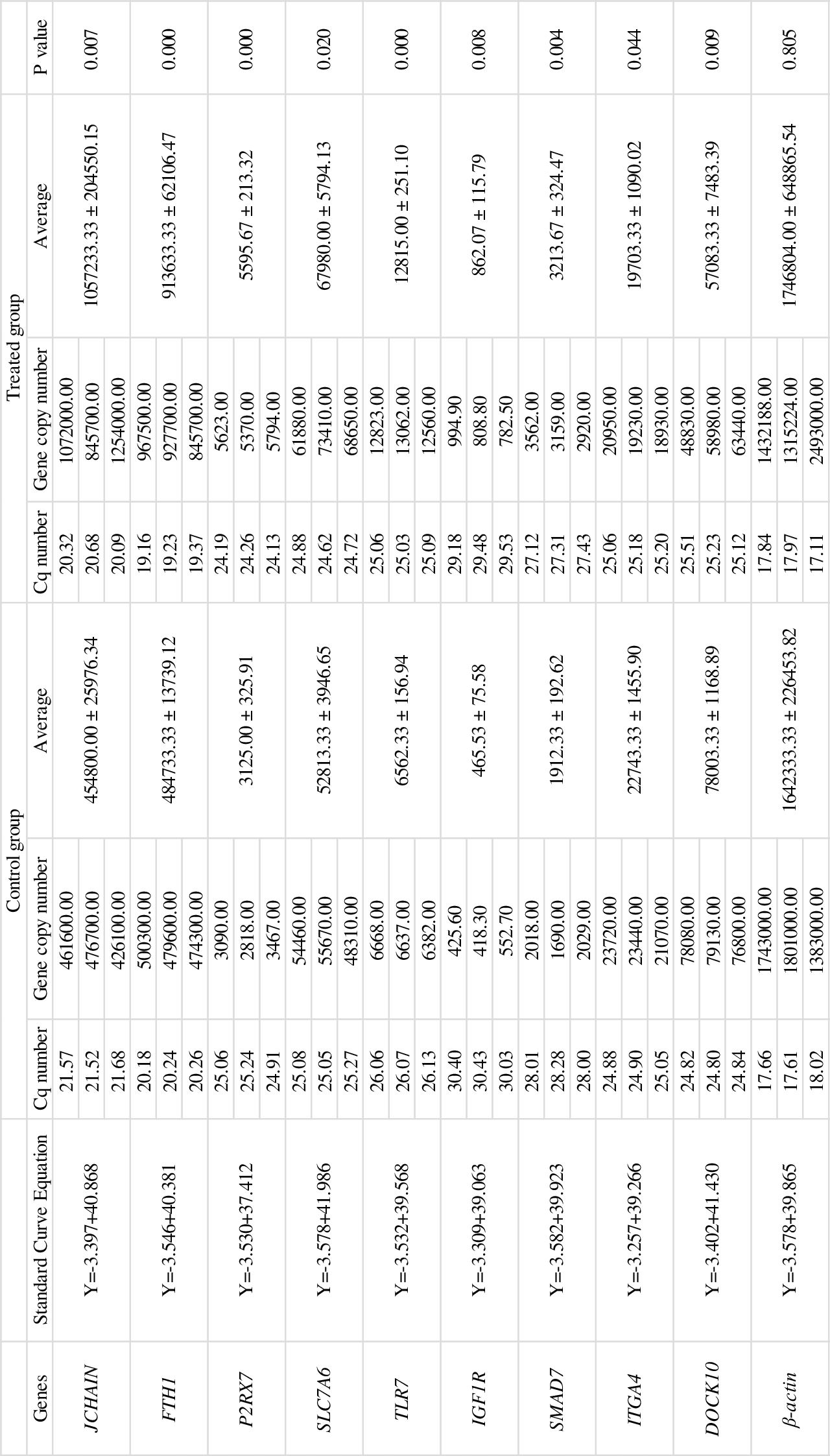

Supplement: S13 Fig — P-value is calculated by SPSS 23.0 software. (TIF) [file pone.0226829.s013.tif]
