# Supplementary material for: Screening of differentially expressed immune-related genes from spleen of broilers fed with probiotic Bacillus cereus PAS38 based on suppression subtractive hybridization
Source: PLoS One. 2019 Dec 23;14(12):e0226829. doi: 10.1371/journal.pone.0226829 (PMC6927618; doi:10.1371/journal.pone.0226829)
Supplement: S1 Table — (PDF) [file pone.0226829.s014.pdf]

Medium Components	Percentage %
Corn flour	2.0 %
Fish meal	1.0 %
Soybean flour	1.0 %
Oil cake	1.0 %
Bran	1.0 %
Nacl	0.5 %
Agar	2.0 %
Distilled water	1 L
PH value	7.0-7.5
