# Supplementary material for: Screening of differentially expressed immune-related genes from spleen of broilers fed with probiotic Bacillus cereus PAS38 based on suppression subtractive hybridization
Source: PLoS One. 2019 Dec 23;14(12):e0226829. doi: 10.1371/journal.pone.0226829 (PMC6927618; doi:10.1371/journal.pone.0226829)
Supplement: S2 Table — (PDF) [file pone.0226829.s015.pdf]

Item	Forward library	Reverse Library
Total sequencing clones	129	155
Low quality sequences	24	59
Unmatched sequences	11	7
Unannotated protein sequences	2	1
Repeat sequences	29	32
Effective gene sequences	63	56
