# Supplementary material for: Screening of differentially expressed immune-related genes from spleen of broilers fed with probiotic Bacillus cereus PAS38 based on suppression subtractive hybridization
Source: PLoS One. 2019 Dec 23;14(12):e0226829. doi: 10.1371/journal.pone.0226829 (PMC6927618; doi:10.1371/journal.pone.0226829)
Supplement: S1 Raw images — (PDF) [file pone.0226829.s016.pdf]

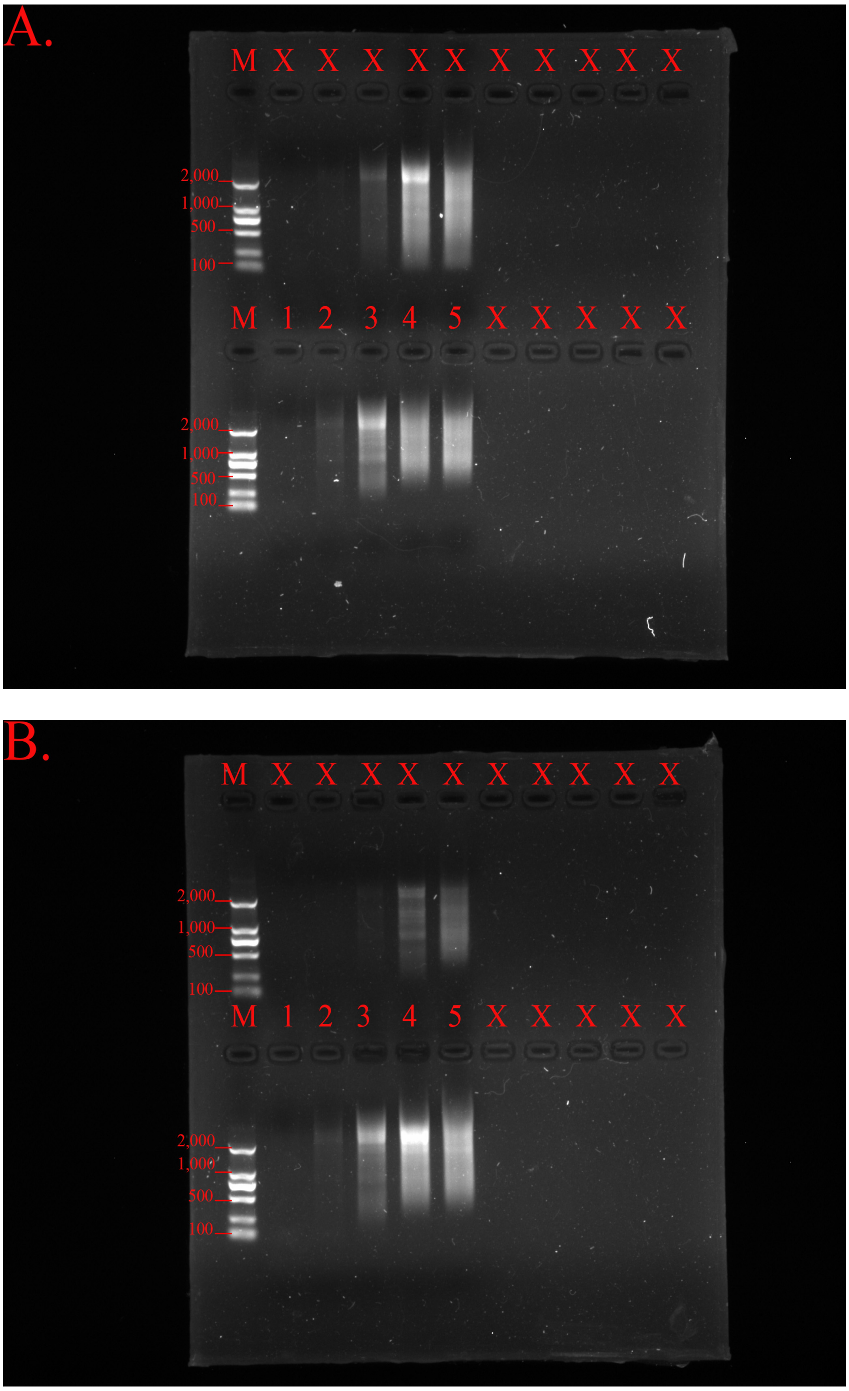

S1 Fig. Agarose electrophoresis analysis of optimal cycles of double-stranded cDNA. Electrophoresis with agarose of 1.2% concentration. The images were generated by the Gel imaging system Gel Doc™ XR+. (A) Treated group. (B) Control group. M represents DNA Marker 2000. The numbers 1, 2, 3, 4 and 5 represent 18, 21, 24, 27 and 30 PCR cycles respectively. Fig 1A was generated by S1A Fig, and Fig 1B was generated by S1B Fig. (Special note: The top row of the gels images in S1A Fig and S1B Fig were the data of our other treated groups that excluded in the manuscript. Therefore, when we cropped images, we did not include them)

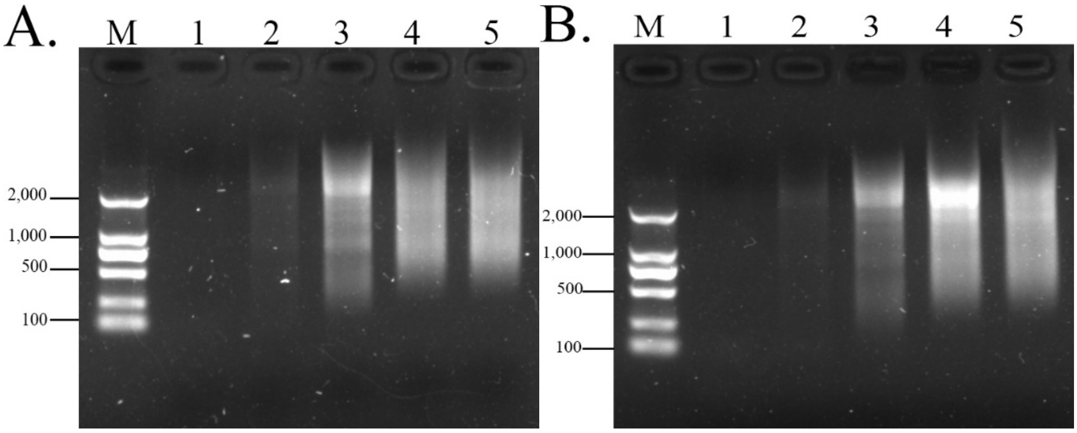

Fig 1. Agarose electrophoresis analysis of optimal cycles of double-stranded cDNA. Electrophoresis with agarose of 1.2% concentration. The images were generated by the Gel imaging system Gel Doc™ XR+. (A) Treated group. (B) Control group. M represents DNA Marker 2000. The numbers 1, 2, 3, 4 and 5 represent 18, 21, 24, 27 and 30 PCR cycles respectively.
