# Supplementary material for: Screening of differentially expressed immune-related genes from spleen of broilers fed with probiotic Bacillus cereus PAS38 based on suppression subtractive hybridization
Source: PLoS One. 2019 Dec 23;14(12):e0226829. doi: 10.1371/journal.pone.0226829 (PMC6927618; doi:10.1371/journal.pone.0226829)
Supplement: S2 Raw images — (PDF) [file pone.0226829.s017.pdf]

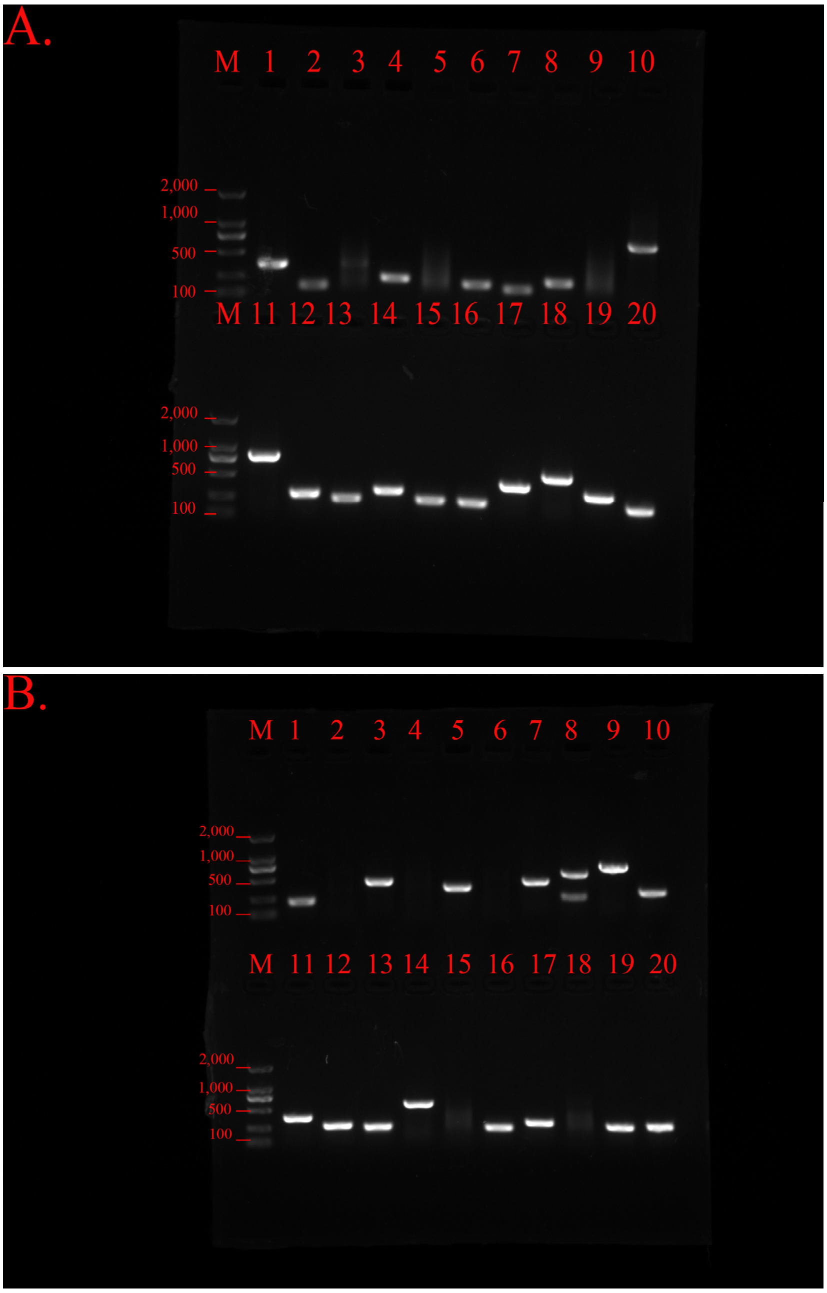

S2 Fig. Detection of inserted fragments by bacteria liquid PCR. Electrophoresis with agarose of 1.2% concentration. The images were generated by the Gel imaging system Gel Doc™ XR+. (A) Treated group. (B) Control group. M represents DNA Marker 2000. The numbers 1, 2, 3, et al. represent different bacterial clones. Fig 2A was generated by S2A Fig, and Fig 2B was generated by S2B Fig.

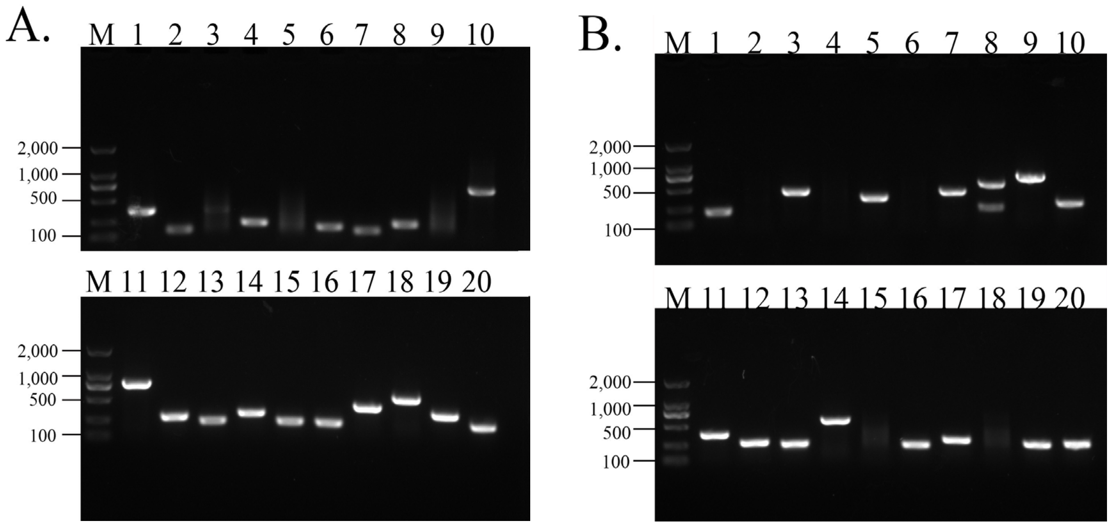

Fig 2. Detection of inserted fragments by bacteria liquid PCR. Electrophoresis with agarose of 1.2% concentration. The images were generated by the Gel imaging system Gel Doc™ XR+. (A) Treated group. (B) Control group. M represents DNA Marker 2000. The numbers 1, 2, 3, et al. represent different bacterial clones.
